# Use of case reports and the Adverse Event Reporting System in systematic reviews: overcoming barriers to assess the link between Crohn’s disease medications and hepatosplenic T-cell lymphoma

**DOI:** 10.1186/2046-4053-2-53

**Published:** 2013-07-05

**Authors:** Saranya A Selvaraj, Elizabeth Chairez, Lisa M Wilson, Mark Lazarev, Eric B Bass, Susan Hutfless

**Affiliations:** 1 Johns Hopkins University School of Medicine, 733 North Broadway, Baltimore, MD 21205-2196, USA; 2 Valley Medical Center, 400 South 43rd Street, Renton, WA 98055-5010, USA; 3 Johns Hopkins University Bloomberg School of Public Health, 615 North Wolfe Street, Baltimore, MD 21205, USA; 4 University of Maryland Medical Center, 22 South Greene Street, Room N3E09, Baltimore, MD 21201-1595, USA

**Keywords:** Crohn’s disease, Hepatosplenic T-cell lymphoma, Causality assessment, Adverse event reporting, Case reports, Systematic reviews

## Abstract

**Background:**

To identify demographic and clinical characteristics associated with cases of hepatosplenic T-cell lymphoma (HSTCL) in patients with Crohn’s disease, and to assess strength of evidence for a causal relationship between medications and HSTCL in Crohn’s disease.

**Methods:**

We identified cases of HSTCL in Crohn’s disease in studies included in a comparative effectiveness review of Crohn’s disease medications, through a separate search of PubMed and Embase for published case reports, and from the Food and Drug Administration (FDA) Adverse Event Reporting System (AERS). We used three causality assessment tools to evaluate the relationship between medication exposure and HSTCL.

**Results:**

We found 37 unique cases of HSTCL in patients with Crohn’s disease. Six cases were unique to the published literature and nine were unique to AERS. Cases were typically young (<40 years of age) and male (86%). The most commonly reported medications were anti-metabolites (97%) and anti-tumor necrosis factor alpha (anti-TNFa) medications (76%). Dose and duration of therapy were not consistently reported. Use of aminosalicylates and corticosteroids were rarely reported, despite the high prevalence of these medications in routine treatment. Using the causality assessment tools, it could only be determined that anti-metabolite and anti-TNFa therapies were possible causes of HSTCL in Crohn’s disease based on the data contained in the case reports.

**Conclusion:**

Systematic reviews that incorporate case reports of rare lethal events should search both published literature and AERS, but consideration should be given to the limitations of case reports. In this study, establishing a causative effect other than ‘possible’ between anti-metabolite or anti-TNFa therapies and HSTCL was not feasible because case reports lacked data required by the causality assessments, and because of the limited applicability of causality assessment tools for rare irreversible events. We recommend minimum reporting requirements for case reports to improve causality assessment and routine reporting of rare life-threatening events, including their absence, in clinical trials to help clinicians determine whether rare adverse events are causally related to a medication.

## Background

Crohn’s disease is an idiopathic, chronic, inflammatory bowel disease that affects the gastrointestinal tract. An estimated 565,000 to 720,000 people in the United States have Crohn’s disease [1]. In the 1950s, corticosteroids and sulfasalazine were adopted as the first immunosuppressive treatments for Crohn’s disease, followed by the anti-metabolites, 6-mercaptopurine and azathioprine, in the 1960s [2]. The twenty-first century has brought with it the first biologic agent for Crohn’s disease, infliximab, a monoclonal antibody against tumor necrosis factor alpha (anti-TNFa). Adalimumab and certolizumab pegol are the other approved anti-TNFa agents.

Hepatosplenic T-cell lymphoma (HSTCL) is a rare and often fatal outcome associated with Crohn’s disease. A boxed warning was issued in 2006 for an association between infliximab and HSTCL. As of October 2011, the label for infliximab reads:

‘Postmarketing cases of fatal hepatosplenic T-cell lymphoma (HSTCL) have been reported in patients treated with TNF blockers including *infliximab*. All *infliximab* cases were reported in patients with Crohn’s disease or ulcerative colitis, the majority of whom were adolescent or young adult males. All had received azathioprine or 6-mercaptopurine concomitantly with *infliximab* at or prior to diagnosis (brand name changed to generic in italics)’ [3].

Despite increasing concerns about the use of anti-TNFa medications, there is no definitively established causal mechanism for HSTCL. Risk factors for HSTCL are thought to include young age, male gender, Crohn’s disease, and renal transplantation [4]. However, HSTCL has occurred in the absence of immunosuppressive treatment and immunodeficiency [5]. Symptoms of HSTCL include fever, cytopenias, and an enlarged spleen and liver [4]. Because of the rarity of HSTCL, cases are unlikely to be identified in trials. Case reports leading to better understanding of Crohn’s disease patients who experience HSTCL may help to identify those patients at increased risk. Causality assessment tools developed for case reports can then be used to determine the likelihood that a medication is causally associated with HSTCL.

We aimed to identify demographic and clinical characteristics and medication histories associated with HSTCL in Crohn’s disease cases published in the peer-reviewed literature or reported to the Food and Drug Administration (FDA) Adverse Event Reporting System (AERS) database. We used three different causality assessment tools to assess the strength of evidence supporting a causal relationship between medication exposures and HSTCL in Crohn’s disease. This project was performed as part of a comparative effectiveness review of treatments for Crohn’s disease [6]. We will also discuss the implications of our findings for the use of case reports in systematic reviews.

## Methods

### Literature search and identification of cases from the published literature

PubMed and Embase were queried on 25 January 2011 using predetermined search strings that included the terms ‘Crohn’s disease,’ ‘inflammatory bowel disease,’ and ‘hepatosplenic T cell lymphoma’ (see full search strings in Additional file 1: Table S1). We included all study types with human patients. Studies were excluded if they were not written in English or if they did not include patients with Crohn’s disease who had developed HSTCL. Additionally, all studies that met the inclusion criteria for the original systematic review were included if they specifically mentioned HSTCL. We also performed a hand-search of references in relevant articles. To avoid double counting of cases that had been reported multiple times in the literature, we checked the footnotes and references, as well as the demographic and clinical characteristics.

### Search of the Food and Drug Administration (FDA) Adverse Event Reporting System (AERS) database and identification of AERS cases

The FDA AERS database was searched for all reported cases of HSTCL from January 2004, the first year data is available online, through December 2010. Only cases that had Crohn’s disease listed as an indication for therapy were included. To avoid double counting of cases reported by multiple sources (such as by a treating physician and a pharmaceutical company), the case entries were matched by case number. If the case number did not match but the report had identical information for three of five criteria (age at diagnosis, date of death, diagnosis date, reporting country, sex), then this entry was reported as a unique case, similar to previous efforts to avoid duplicates using AERS [7].

### Matching cases from the published literature with AERS cases

We sought to distinguish cases reported in both the published literature and AERS from those cases reported in only one source, using the following information: age at HSTCL diagnosis; date of HSTCL diagnosis or date of publication or AERS report when HSTCL date not reported; sex; medication history; reporting country (AERS) or country of origin (published literature); and date of death (AERS) or survival status or months of survival (published literature) (see Additional file 2: Table S2). If a case report from AERS and the literature matched on three of the five criteria, then the case was reported as a single unique case reported in both sources. We then identified cases reported in the published literature or AERS with sufficient information to consider them a unique case reported in only one source. Finally, some entries did not have sufficient details to determine if they were a unique case. We recorded the demographics of entries with insufficient reporting, but excluded the entries with insufficient reporting from the causality assessment.

### Causality assessment

To evaluate the evidence supporting the association of medication exposures with HSTCL, we used causality assessment tools developed by the World Health Organization (WHO), Naranjo *et al.*, and Kramer *et al. *[8-10]. Two evaluators (EC and SS) independently calculated the causality assessment score for each medication used in each case and then met to discuss and resolve by consensus differences between the scores.

Table 1 compares the characteristics and content of the three causality assessments. The Naranjo and Kramer assessments produced numerical scores that corresponded with causal categories of definite, probable, possible, and unlikely/doubtful. The WHO assessment assigns cases to the same categories as well as two additional categories of conditional/unclassified and unassessable/unclassifiable.

**Table 1:** Characteristics of published instruments assessing causal relationship between medication exposure and adverse events

	**Naranjo**	**Kramer**	**WHO**
Numerical range	−4 to +13	−7 to +7	n/a
Format	Ten questions answered ‘yes,’ ‘no,’ or ‘don’t know,’ each with a numerical value; numbers summed for final score. Higher scores indicate greater likelihood of causality.	An algorithm with six separate axes/flowcharts. Each axis contributes points to the final score. Higher scores indicate greater likelihood of causality.	Contains six categories; the user selects the one for which most criteria are met.
Distinguishes irreversible adverse events from other adverse events?	No	Yes	No
Addresses methods used to confirm adverse event?	Yes	No	No
Asks about serum medication levels?	Yes	Yes	No
Considers prior experience with medication?	Yes	Yes	Yes
Considers alternate etiologies?	Yes	Yes	Yes
Questions about rechallenge?	Yes	Yes	Yes
Questions about dechallenge?	Yes	Yes	Yes

n/a, not applicable; WHO, World Health Organization.

## Results

### Search results and identification of unique cases

**Figure 1:**
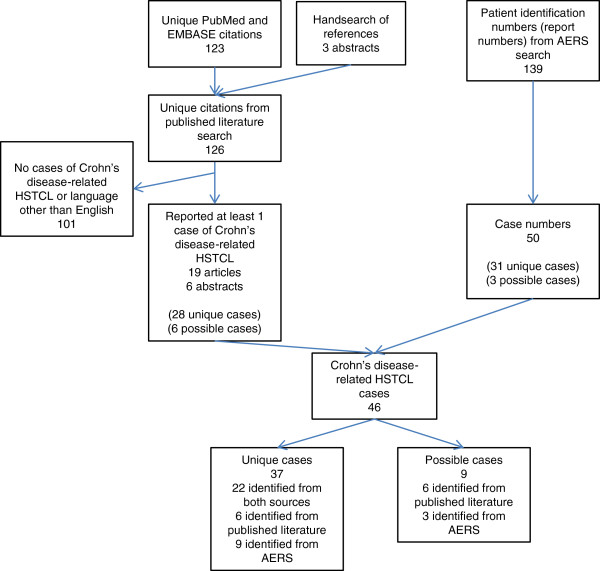
**Identification of unique cases by PubMed and Embase searches.** AERS, Adverse Event Reporting System; HSTCL, hepatosplenic T-cell lymphoma.

Of the 123 citations identified by the PubMed and Embase searches, 19 case reports or case series [5,11-28] and three conference-presentation abstracts [29-31] met the inclusion criteria of reporting at least one Crohn’s disease-related HSTCL case (Figure 1). Three additional conference-presentation abstracts were identified through a hand-search of the references of the included articles [32-34]. No comparative studies that met the inclusion criteria for the systematic review reported a case of HSTCL. One prospective study reported specifically that no cases of HSTCL were observed [35]. Thirty-four cases were identified from the published literature with 28 having sufficient information to be considered unique cases.

A search of the publicly available AERS data files yielded 139 patient identification numbers representing 50 unique case numbers, which after matching yielded 34 cases with 31 having sufficient information to be considered unique.

After matching unique cases from the published literature with the unique cases from AERS, 37 total cases were considered unique (22 from both sources, six from the published literature only, and nine from AERS only; Additional file 1: Table S1). Thirty-six HSTCL cases were considered unique based on age, sex, and medication exposures. An additional case was considered unique based on the case’s use of cyclosporine even though age at HSTCL diagnosis and sex were not reported [32]. Nine cases had insufficient reporting (Additional file 2: Table S2).

### Demographics, presenting symptoms and survival

The 37 unique patients were young (84% of patients were younger than age 40 years) and male (86%; Table 2). Patients had Crohn’s disease for a mean of 10 years prior to their diagnosis of HSTCL (range: 4 to 35 years). All patients reporting any symptoms presented with hepatosplenomegaly or splenomegaly (100%), and approximately one-half presented with fever (47%) or cytopenia (58%). The median length of survival among the 26 cases with information was 7 months (range: 5 days to 9.7 years). HSTCL resulted in death for 65% of patients. Survival was not reported for 30% of cases and two patients were alive at the time of the case report: one patient at 3 months after receiving chemotherapy and a bone marrow transplant, and another patient at 20 months after HSTCL diagnosis.

**Table 2:** Demographic and clinical characteristics of reported Crohn’s disease patients with hepatosplenic T-cell lymphoma (HSTCL)

	**Unique cases (n = 37)**	**Cases with insufficient reporting**^**a **^**(n = 9)**
**Age at HSTCL diagnosis, years**	**n = 36**	**n = 0**
Mean	30	-
Median	26	-
Range	12 to 79	-
**Disease duration, years**	**n = 16**	**n = 0**
Mean	10	-
Median	6	-
Range	4 to 35	-
**Sex, n (%)**	**n = 36**	**n = 6**
Female	5 (14%)	1 (17%)
**Survival, n (%)**	**n = 26**	**n = 4**
Died	24 (92%)	4 (100%)
Survived	2 (8%)	0 (0%)
**Physical examination and laboratory abnormalities at time of HSTCL diagnosis, n (%)**	**n = 19**	**n = 1**
Hepatosplenomegaly or splenomegaly	19 (100%)	1 (100%)
Fever	9 (47%)	-
Cytopenia of any type	11 (58%)	-
Altered liver enzymes and/or LDH	5 (26%)	-

^a^Insufficient reporting on demographic information prevented us from identifying if the cases were unique. With each patient characteristic heading, the adjacent ‘n = x’ cells indicate the number of cases that reported on that particular demographic. The percentages calculated for sex, survival, and physical examination/laboratory abnormalities use this ‘n’ as the denominator. HSTCL*,* hepatosplenic T-cell lymphoma, LDH, lactate dehydrogenase.

Information about the patient’s race, Crohn’s disease location, behavior, and severity was infrequently reported in published case reports and case series. This information is not requested in the AERS case report form. Race was reported for two patients, both of whom were white [15,25]. Five patients had information on disease location: one patient had ileal disease [25], two patients had ileocolonic disease [12,17], one patient had ileal and perianal disease [15], and one patient had perianal disease [32]. Disease behavior was reported for one patient who had inflammatory disease [14].

### Medication history

**Figure 2:**
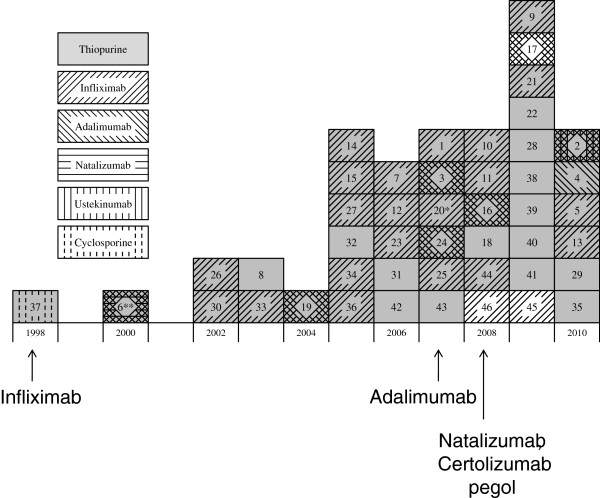
**Timeline of medication approval by the Food and Drug Administration (FDA) and occurrence of hepatosplenic T-cell lymphoma (HSTCL).** Includes 37 unique cases and nine cases with insufficient reporting, for a total of 46 cases. Each box includes a unique case by year the case was reported. The horizontal axis indicates the year the case was diagnosed. The numbers in each square are ordered oldest to youngest age at HSTCL diagnosis. They match up to the numbers with detailed case information in Additional file 2: Table S2. Vertical arrows indicate years that the particular medications were approved by the FDA. Ustekinumab is not approved by the FDA for Crohn’s disease. The numbers within each box refer to case numbers in Additional file 2: Table S2. Patients were reported through 25 January 2011 (published) and December 2010 (AERS). *Date of case reported as 2007 to 2008. **Date of case reported as 2000 to 2009. AERS, Adverse Event Reporting System; FDA, Food and Drug Administration; HSTCL, hepatosplenic T-cell lymphoma.

The timeline in Figure 2 displays the report of new HSTCL cases in patients with Crohn’s disease from 1998 through 2010, including the 37 unique cases and the nine cases with insufficient information. Case exposure patterns for biologic medications were similar to the timeline of FDA approval for Crohn’s disease: 96% of biologic exposures among the 37 unique cases involved infliximab (approved in 1998), 29% adalimumab (approved in 2007), 4% natalizumab (approved in 2008), 4% ustekinumab (not approved as of December 2012), and thus far no affected patients had certolizumab pegol exposure (approved in 2008). All cases that used a biologic had also used infliximab, with the exception of one case who used adalimumab only. The use of concurrent medications or medications at the time of HSTCL diagnosis cannot be summarized because this information was not uniformly reported.

The most frequently reported medications used were anti-metabolites and anti-TNFa agents (Table 3). Thirty-six unique cases (97%) used an anti-metabolite and 28 cases (76%) used an anti-TNFa. Eight cases had used an anti-metabolite without an anti-TNFa agent. One case had used cyclosporine and an anti-metabolite. Of the patients who used anti-TNFa therapy, 27 cases (96%) had also used an anti-metabolite, but not necessarily at the same time. One patient who used anti-TNFa therapy without anti-metabolites was reported in the AERS database. This patient had infliximab and adalimumab exposure (Additional file 2: Table S2).

**Table 3:** Medications used to treat Crohn’s disease prior to the diagnosis of hepatosplenic T-cell lymphoma (HSTCL) among unique cases

**Medication**	**Number of cases with exposure reported, n (%) (n = 37)**	**Mean cumulative dose, mg (minimum - maximum)**^**a**^	**Mean duration of use, years (minimum - maximum)**^**a**^	**Mean number of infusions or injections (minimum - maximum)**^**a**^
**Biologics**	28 (76%)			
Adalimumab	8 (22%)	920 (800 to 1040)	1.5 (120 days to 2.6 years)	11.5 (10 to 13)
		n = 2	n = 2	n = 2
Infliximab^**a**^	27 (73%)	41 (10 to 120) mg/kg	1.8 (1 day to 6 years)	9 (1 to 24)
		n = 7	n = 12	n = 17
Natalizumab	1 (3%)	NR	NR	3
Ustekinumab	1 (3%)	NR	NR	NR
Certolizumab pegol	0			
**Anti-metabolites**	365 (97%)			
6-mercaptopurine	20 (54%)	94,508 (3,900 to 212,160)	4.8 (39 days to 8 years)	
		n = 4	n = 10	
Azathioprine	23 (62%)	192,108 (1,450 to 301,125)	5.8 (39 days to 13.5 years)	
		n = 3	n = 17	
**Aminosalicylates**	15 (41%)			
Balsalazide	1 (3%)	NR	NR	
Mesalamine	13 (35%)	NR	5 (n = 1)	
Sulfasalazine	2 (5%)	NR	10 (n = 1)	
**Corticosteroids**	22 (59%)			
Budesonide	2 (5%)	NR	NR	
Hydrocortisone	1 (3%)	NR	NR	
Prednisone	14 (38%)	NR	NR	
Prednisolone	5 (14%)	NR	13 (n = 1)	
Corticosteroid	4 (11%)	NR	10 (n = 1)	
**Other medications**^**b**^	15 (41%)			
Antibiotics^c^	8 (22%)	NR	NR	
Cyclosporine	1 (3%)	NR	NR	

^a^Among those that reported this data. Cumulative dose excludes cases that only provided mg/kg dosing but did not provide patient weight in kilograms and those cases which reported a daily dose but did not report duration of treatment. ^b^Excluding vitamin/mineral supplements. ^c^The antibiotics used included ciprofloxacin, doxycycline, metronidazole, nitrofurantoin, and piperacillin/tazobactam. HSTCL, hepatosplenic T-cell lymphoma; NR, not reported.

Daily dose, duration, or cumulative dose of therapy were not consistently reported (Table 3). The majority of ‘mean doses’ were based on one to two patients. Infliximab was the only medication where measures of dose and duration were consistently provided. For the anti-metabolites, information on duration of treatment was also frequently provided, but information on daily or cumulative dose less so. Aminosalicylate and corticosteroid exposures were less commonly reported, despite the frequent use of aminosalicylates to treat colonic inflammatory disease and the use of corticosteroids to treat disease flares.

### Medication causality assessment

Table 4 summarizes causality assessments based on the 37 unique cases. Using three causality assessment tools, the majority of medications received a score consistent with ‘possible’ cause. All three tools had criteria for rechallenge and dechallenge tests that could not be performed for an irreversible outcome like lymphoma, thus lowering the score. The tool of Kramer *et al.* included criteria for the incidence of HSTCL, which has not been estimated for Crohn’s disease, thus lowering the score.

**Table 4:** Results of the published instruments assessing causal relationship between medication exposure and adverse events when applied to patients in this case series

**Medication(s)**	**Naranjo score**	**Kramer score**	**WHO score**
**Biologics**			
Adalimumab	Possible	Possible	Possible
Certolizumab pegol	No use reported in any case report		
Infliximab	Possible	Possible	Possible
Natalizumab	Possible	Possible	Possible
Ustekinumab	Possible	Possible	Possible
**Anti-metabolites**			
6-mercaptopurine	Possible	Possible	Possible
Azathioprine	Possible	Possible	Possible
**Aminosalicylates**			
Balsalazide	Possible	Unlikely	Possible
Mesalamine	Possible	Unlikely	Possible
Sulfasalazine	Possible	Unlikely	Possible
**Corticosteroids**			
Budesonide	Possible	Unlikely	Possible
Hydrocortisone	Possible	Unlikely	Possible
Prednisone	Possible	Unlikely	Possible
Prednisolone	Possible	Unlikely	Possible
Corticosteroid unspecified	Possible	Unlikely	Possible
**Other medications**			
Cyclosporine	Possible	Possible	Possible
Metronidazole	Possible	Unlikely	Possible
Nitrofurantoin	Possible	Unlikely	Possible
Piperacillin/tazobactam	Possible	Unlikely	Possible
Doxycycline	Possible	Unlikely	Possible
Ciprofloxacin	Possible	Unlikely	Possible

Possible, all cases reporting that medication received a score of ‘possible’ using that particular causality assessment; unlikely, all cases reporting that medication received a score of ‘unlikely’ using that particular causality assessment. WHO, World Health Organization.

Scores calculated using the Naranjo method ranged from 1 to 2 points, corresponding with ‘possible’ causation (1 to 4 points) for each patient who used each medication. The items that contributed to the score for most patients included a point for administration prior to the development of HSTCL, and documentation of histopathology to confirm HSTCL diagnosis. All patients and medications lost one point because HSTCL has been reported in the absence of medications [5].

Scores calculated using the Kramer method did vary between medication classes. Anti-metabolites, biologics, and cyclosporine were determined to be a ‘possible’ cause of HSTCL (0 to 3 points). All other medications scored −1 point, determined to be an ‘unlikely’ cause (< 0 points). The variation in scores was due to previous reports of adverse reactions with the medication and availability of the medication. Anti-metabolites and cyclosporine were scored 0 points because they have been linked to other lymphomas. Natalizumab and ustekinumab were scored 0 points because they have been available for less than 20 years, so all adverse reactions have not yet been described. Anti-TNFa medications were scored 1 point because they have been associated with other lymphomas and have been available for less than 20 years. Antibiotics and corticosteroids received scores of −1 point because the medications have been available for long enough for most adverse reactions to have been previously reported and they have not been associated with lymphoma.

Assessments of causality using the WHO method provided determination of ‘possible’ causation. Because the time required for development of HSTCL is unknown, any medication administered before HSTCL diagnosis was considered to be within a ‘reasonable’ time period leading up to HSTCL development. Because HSTCL could have been explained by another medication or Crohn’s disease itself, each medication used for each patient met the criterion for an alternative etiology.

## Discussion

All medications were possibly related to HSTCL according to at least one causality assessment tool. Anti-metabolites, biologics, and cyclosporine were possibly related to HSTCL according to all three causality assessment tools. Despite the possible causal relationship found across three different tools for these medications, only the anti-TNFas carry a boxed warning for HSTCL. The label for azathioprine includes a boxed warning for malignancy with mention of HSTCL, and mercaptopurine’s label mentions HSTCL in the non-boxed warnings. The label for cyclosporine mentions the risk of lymphoma in the boxed warnings, but not HSTCL specifically. The labels for natalizumab and ustekinumab do not mention lymphoma. The most common demographic risk factors were male sex and younger age, similar to previous studies [22,26,27]. No comparative study from the main systematic review included a case of HSTCL, and only one study specifically mentioned that no HSTCL cases were observed.

The methods guides for systematic reviews of the Evidence-based Practice Center (EPC) Program and the Cochrane Collaboration recommend using case series in some instances [36-38]. Based on the importance of HSTCL to patients, HSTCL’s high mortality and the rarity of HSTCL, we performed a separate search of the published literature for HSTCL case reports and case series, and searched AERS as part of a systematic review for treatments of Crohn’s disease. Our rationale for including cases is consistent with the recommendations of the methods guides. Other systematic reviews have included case reports and case series identified from the literature or cases from AERS based on similar rationale [39-43]. In contrast to many previous systematic reviews, we searched both case sources. Searching AERS yielded nine cases not identified in the published literature. However, because the majority of cases were identified in both AERS and the published literature, we had to create a process to identify the overlapping cases.

Insufficient reporting prevented us from determining the uniqueness of all identified cases. A few case reports failed to include patient age or sex. Many cases identified as unique did not include information on medication dose, frequency, or dates of use, even though reporting systems such the FDA AERS request this demographic and medication data on their submission forms [44]. Incomplete reporting is also prevalent in case reports on other types of adverse medication events, and calls have been made for a standardized list of case report guidelines similar to the checklists created by organizations for other types of observational studies [45]. Other agencies beside the FDA also request specific demographic, clinical, laboratory, and medication information in adverse event reports [46]. Nevertheless, no standard checklist exists for case reporting, and journal requirements for case report content are variable [47].

The usefulness of case reports to identify potential adverse medication events will be enhanced by a standardized checklist for case reporting. Such a checklist should incorporate information commonly requested for adverse event reports and causality assessment, including: basic demographics, documentation of the use and timing of use of the individual and concomitant medications used to treat the condition (including absence of use), all other medications used by the patient, rechallenge and dechallenge information (including absence), laboratory results (including serum medication levels), and diagnostic tests used to confirm the adverse event. Reporting the complete list of medications used by the cases, rather than focusing on medications previously associated with the adverse events, as was common in the case reports we identified, will also aid in the understanding of drug interactions and the adverse event. Modifying the AERS database to require these elements for submission and standardizing the information in case reports submitted to journals will help to match AERS cases to the published cases, identify trends by items like race that are not consistently reported, and improve causality assessments. The suggested modifications are consistent with the Institute for Safe Medication Practices’s (ISMP) recommendation to update AERS to improve the quality of reporting so that the FDA can make more targeted safety warnings [48,49].

Using the causality assessment tools for an irreversible and almost uniformly fatal condition such as HSTCL was challenging. Based on available data, we could not make any causal assessments other than ‘possible’ for all medications. Some elements, such as rechallenge with medication, are not possible for events that rapidly progress to mortality. Improvement of symptoms for cancer with dechallenge is also rare, although regression of lymphoma with withdrawal of azathioprine has been documented [50]. Other items in the causality tools required more complete reporting, such as serum medication levels or details of the case confirmation, were not available for all cases. Better case reporting and understanding how to modify causality tools for use with irreversible adverse events will assist in making causality assessments for rare events from case reports. For events such as HSTCL that likely have a complex, multifactorial etiology that may develop over months to years, reporters should be prompted to include more detailed information on patient medical history (few of the reports had details on Crohn’s disease severity and behavior), family history, and the absence of use or past use of certain medications commonly used to treat the underlying disease. This data could be requested as part of the FDA’s existing enhanced safety surveillance of TNFa-blockers and pediatric and young adult malignancy, or even in routine submission of MedWatch forms for adverse events such as malignancy [51]. Journals can also request that authors provide this history when submitting case reports on malignancies that are possibly associated with medications. Routine reporting of this additional information may accelerate identification of other medications and risk factors associated with the adverse event. Causal assessments can then be modified to incorporate relevant biology and epidemiology and used to prioritize which safety events should be studied further to estimate a rate by medication use.

When possible causality has been established from case reports, trials and observational studies can contribute information to estimate a rate of disease by medication use. Trials and prospective studies may begin collecting information on safety events like HSTCL. If trials and observational studies report the occurrence of these rare events and details of the active or passive collection process, the rate of HSTCL can be estimated. For trials and comparative medication studies, a rate by medication use can be calculated by pooling the studies. Understanding the rate of disease, which is not possible from case reports, can help patients and their caretakers make better treatment decisions. Trials and observational studies should also attempt to collect specific details about the events. For example, the TREAT Registry, a Phase IV study for infliximab, reported a case of fatal peripheral T-cell lymphoma with infliximab and immunomodulator use within 6 months of death [52]. Readers cannot determine whether the T-cell lymphoma was HSTCL because details were not provided in the main text of the publication or the supplemental table. By providing more detailed information about the cause of death, particularly when a death may be due to a black box warning-related event, Phase IV studies could help to improve understanding of the rate of life-threatening events such as HSTCL in these important safety studies.

## Conclusions

Consistent with FDA safety warnings, we confirmed that anti-metabolites, anti-TNFas, and cyclosporine have a possible causal association with HSTCL. We were unable to assign any score other than ‘possible’ using existing causality assessments because of limitations in data reported in case reports and difficulty in obtaining rechallenge and dechallenge data for a fatal event such as HSTCL. Minimum reporting requirements for peer-reviewed literature and AERS will help facilitate the use of case reports to identify adverse events and assess causality. Reporting the absence of HSTCL in trials and cohort studies will provide information to estimate a rate of HSTCL occurrence by medication use. Intentional assessment will be particularly useful in studies that include children and young adults who appear to be at a greater risk of HSTCL. Our findings provide support for the development and adoption of case report guidelines, a topic that will be discussed at the next International Congress on Peer Review and Biomedical Publication [53]. Standards for collecting additional patient history in case reports and modifying causality assessment tools for irreversible events such as a fatal lymphoma will assist in making causal assessments using case reports.

## Abbreviations

AERS: Adverse event reporting system; AHRQ: Agency for Healthcare Research and Quality; anti-TNFa: anti-tumor necrosis factor alpha; EPC: Evidence-based Practice Center; FDA: Food and Drug Administration; HSTCL: Hepatosplenic T-cell lymphoma; ISMP: Institute for Safe Medication Practices; NCRR: National Center for Research Resources; NIH: National Institutes of Health; WHO: World Health Organization.

## Competing interests

The authors do not have any competing interests to declare.

## Authors’ contributions

SS and EC reviewed search results, performed data extraction and analysis, and applied the causality assessment tools. SS, EC, LW, and SH drafted the manuscript. EB was responsible for overseeing all work done on this project by the Evidence-based Practice Center (EPC) as part of its contract with the Agency for Healthcare Research and Quality (AHRQ). All authors contributed to the conception and design of the study, and read, revised, and approved the final manuscript.

## Supplementary Material

Additional file 1: Supplemental Table 1 Search strings used to identify cases in PubMed and Embase. Click here for file

Additional file 2: Supplemental Table 2 Case series: characteristics of individual cases identified in the literature and Adverse Event Reporting System. Click here for file
