# Supplementary material for: Use of case reports and the Adverse Event Reporting System in systematic reviews: overcoming barriers to assess the link between Crohn’s disease medications and hepatosplenic T-cell lymphoma
Source: Syst Rev. 2013 Jul 5;2:53. doi: 10.1186/2046-4053-2-53 (PMC3710465; doi:10.1186/2046-4053-2-53)
Supplement: Additional file 1: Supplemental Table 1 — Search strings used to identify cases in PubMed and Embase. [file 2046-4053-2-53-S1.docx]

**Supplemental Table 1. Search strings used to identify cases.**

- - *Pubmed: Hepatosplenic T cell lymphoma AND ("Crohn Disease"[mh] OR "Crohn's Disease"[tiab] OR "Crohn Disease"[tiab] OR "Crohns Disease"[tiab] OR (Crohn*[tiab] AND (ileitis[tiab] OR enteritis[tiab] OR ileocolitis[tiab] OR colitis[tiab])) OR "inflammatory bowel diseases"[mh] OR "inflammatory bowel disease"[tiab] OR "inflammatory bowel diseases"[tiab] OR IBD[tiab])*
  - *EMBASE: 'crohn disease'/exp OR 'crohn disease' OR 'inflammatory bowel disease'/exp OR 'inflammatory bowel disease' AND hepatosplenic AND t AND ('cell'/exp OR cell) AND ('lymphoma'/exp OR lymphoma)*
