# Supplementary material for: Use of case reports and the Adverse Event Reporting System in systematic reviews: overcoming barriers to assess the link between Crohn’s disease medications and hepatosplenic T-cell lymphoma
Source: Syst Rev. 2013 Jul 5;2:53. doi: 10.1186/2046-4053-2-53 (PMC3710465; doi:10.1186/2046-4053-2-53)
Supplement: Additional file 2: Supplemental Table 2 — Case series: characteristics of individual cases identified in the literature and Adverse Event Reporting System. [file 2046-4053-2-53-S2.docx]

**Supplemental Table 2. Characteristics of cases identified in the literature and Adverse Event Reporting System.**

|  | **Author Year, Patient # (if from case series), and/or AERS number** | **Age/Sex** | **Medications (cumulative dose/duration)** | **Presenting Symptoms** | **Physical Exam and Lab Findings** | **Outcome (Months to Death)** |
| --- | --- | --- | --- | --- | --- | --- |
|  | UNIQUE HSTCL CASES |  |  |  |  |  |
| **1** | AERS 6445097 | 79/F | IFX, 6MP |  |  | D |
| **2** | AERS 7410990 | 68/F | ADL, IFX, UST, AZA |  |  | U |
| **3** | Biegel 2009, Kotlyar 2010 Pt. 12, AERS 6650567,* 7117443; | 58/M | ADL (800 mg in 10 injections/120 days), IFX (1150 mg in two 5 mg/kg infusions/14 days), AZA (2.5 mg/kg daily/intermittent over 5.5 years), CS, metronidazole, piperacillin, tazobactam, lercanidipine,  piretanide, doxazosin, filgrastim | Fever | F, HSM, P | D (1.5-7.5*) |
| **4** | Thai 2010 Pt. 22 | 58/M | ADL, AZA |  |  | U |
| **5** | AERS 7341031 | 53/M | IFX, 6MP, alendronate sodium, fenofibrate, nadolol, |  |  | U |
| **6** | Kotlyar 2011 Pt. 18, AERS 7068488, 7199651 | 52/M | ADL, IFX (3 infusions/--), NTL (3 infusions/---), AZA (---/13.5 years), PDL, PDE, , nortriptyline, methadone |  |  | D (116) |
| **7** | He 2007, Drini 2008, Shale 2008 Pt. 15, Mackey 2009 Pt. 12, Kotlyar 2009a Pt. 4, Kotlyar 2009b Pt. 14 and 17, Ochenrider 2010 Pt. 17, 25, and 27, Kotlyar 2010 Pt. 3, Thai 2010 Pt. 18, Kotlyar 2011 Pt. 11, AERS 6369868, 6894950 | 39/M | IFX (3 infusions of 5 mg/kg each/90-365 days), AZA (2-2.5 mg/kg daily/7.25 years), PDL (---/13 years) | Malaise, abdominal pain | F, HSM, P, LFT, LDH; Sepsis, Tachycardia, Hypotension, Atypical blood film | D (12-17) |
| **8** | Navarro 2003, Mackey 2007 (text), Zeidan 2007 Pt. 1, Kotylar 2009a Pt. 1, Kotylar 2009b Pt. 19, Moran 2009 Pt. 2, Ochenrider 2010 Pt. 5, Kotlyar 2010 Pt. 5, Thai 2010 Pt. 2, Kotlyar 2011 Pt. 22 | 35/M | AZA (301.125 g/5.6 years), SLZ (---/10 years), CS (---/10 years) | Malaise, night sweats, petheciae on abdomen | HSM, LFT, LDH; abnormal blood smear, elevated β2-microglobulin and CA-125 | D |
| **9** | AERS 7315374, 7417804 | 34/F | IFX (unknown number of 10 mg/kg infusions), AZA |  |  | U |
| **10** | Kotlyar 2011 Pt. 15, AERS 6835594 | 33/M | IFX (---/5 infusions), AZA (---/7 years), MSL, CS |  |  | U |
| **11** | Shale 2008 Pt. 14, Kotylar 2009b Pt. 13, Ochenrider 2010 Pt. 24, Kotlyar 2011 Pt. 19, AERS 6803956 | 31/M | IFX, 6MP (---/3 years), AZA, PDL (5 mg daily/--), , metronidazole |  |  | D |
| **12** | Rosh 2007 Pt. 8, Mackey 2009 Pt. 10, Kotylar 2009b Pt. 10, Ochenrider 2010 Pt. 16, AERS 6179315, 7407778, 6959389, 7064977* | 31/M | IFX (3 infusions of 5 mg/kg each/60 days), AZA (273.75 g/5 years), 6MP* (---/6 years), PDL (5 mg daily/---), metronidazole* |  | HSM | D (3.5) |
| **13** | Thai 2010 Pt. 17 | 31/M | IFX (3 infusions of 5 mg/kg each/---), AZA (---/5 years), PDL, SLZ |  |  | U |
| **14** | Zeidan 2007 (index), Shale 2008 Pt. 13, Mackey 2009 Pt. 11, Kotlyar 2009a Pt. 5, Kotlyar 2009b Pt. 1, Ochenrider 2010 Pt. 3 and 21, Kotlyar 2010 Pt. 2, Thai 2010 Pt. 8, Kotlyar 2011 Pt. 7, AERS 7694051 | 31/M | IFX (300 mg in 1 infusion***^2^/1 day), 6MP (---/6 years), MSL, PDE, | Chills, malaise | F, SM, P, LDH, LFT | D (7) |
| **15** | Mackey 2007/2009 Pt. 1, Rosh 2007 Pt. 10, Shale 2008 Pt. 1, Ochenrider 2010 Pt. 8, Thai 2010 Pt. 14, Kotlyar 2011 Pt. 12, AERS 6240979**, 7392519** | 31**/M | IFX (***^3^/---), 6MP (---/5 years), MSL, PDE |  |  | D (12) |
| **16** | Shale 2008 Pt. 11, Kotlyar 2009b Pt. 12, Ochenrider 2010 Pt. 23, Thai 2010 Pt. 21, Kotlyar 2011 Pt. 14 | 29/M | ADL, IFX (1200 mg/3 infusions), AZA (---/11 years) |  |  | U |
| **17** | AERS 7114026 | 28/M | ADL, IFX (---/6 years), Allopurinol, Famotidine, Lansoprazole, Levothyroxine, Acetominophen |  |  | U |
| **18** | Humphreys 2008, Ochenrider 2010 Pt. 28, Kotlyar 2010 Pt. 8, Kotlyar 2011 Pt. 28 | 27/F | AZA (---/5 years), CS, metronidazole | Fevers, night sweats | HSM, P, LFT | S |
| **19** | Mackey 2009 Pt. 15, Ochenrider 2010 Pt. 20, AERS 6549556 | 25/M | ADL (1040 mg***^4^ in 13 injections/943 days), IFX (1200 mg***^4^ in 3 infusions/60 days), AZA |  | HSM | D* (57) |
| **20** | Kotlyar 2011 Pt. 20, AERS 7138070, 7385131 | 24/M | IFX, 6MP, MSL, PDE |  |  | U |
| **21** | AERS 7411767 | 23/M | IFX, 6MP |  |  | U |
| **22** | Fowler 2010 Pt. 2, Kotlyar 2011 Pt. 36, AERS 7415872, 7428051 | 22/M | 6MP (---/8 years) | Fatigue, night sweats, LUQ tenderness | F, SM | S |
| **23** | Rosh 2007 Pt. 9, Shale 2008 Pt. 12, Kotlyar 2009a/2009b (index) and 2009b Pt. 9, Mackey 2009 Pt. 9, Ochenrider 2010 Pt. 2, 15, and 22, Thai 2010 Pt. 15, Kotlyar 2010 Pt. 14, Kotlyar 2011 Pt. 9, AERS 6188165,* 7696031 | 22/M | IFX (24 infusions of 5 mg/kg each/3.8 years), 6MP (---/^***5^), BLS, MSL, PDE, esomeprazole*, nystatin*, montelukast* |  | HSM, N | D (9.5-16*) |
| **24** | AERS 6447799, 6571268 | 20/M | ADL, IFX (6650 mg in 19 infusions/3.7 years), 6MP (212.16 g/7.75 years), MSL, PDE |  |  | D (5) |
| **25** | Shale 2008 Pt. 16, Mackey 2009 Pt. 13, Kotylar 2009b Pt. 15, Ochenrider 2010 Pt. 18 and 26, Thai 2010 Pt. 20, Kotlyar 2011 Pt. 13, AERS 6507779,* 6673203* | 19/M | IFX (18 10 mg/kg infusions/1.9 years), 6MP (---/2.8 years), PDE, MSL, doxycycline*, allopurinol*, bupropion*, dicyclomine*, loratidine*, pantaprazole*, sertraline*, zolpidem* |  | HSM | D (5) |
| **26** | Mackey 2007/2009 Pt. 5, Rosh 2007 Pt. 4, Shale 2008 Pt. 5, Kotylar 2009b Pt. 5, Ochenrider 2010 Pt. 11, Thai 2010 Pt. 11, Kotlyar 2011 Pt. 5, AERS 3803934* | 19/M | IFX (8050 mg***^6^ in 14 infusions/3 years), AZA(---/6 years), 6MP, PDE, BDE*, MSL**,** nitrofurantoin | Rash | F, HSM, L | D (5*-10.5) |
| **27** | Mackey 2007/2009 Pt. 7, Rosh 2007 Pt. 5, Shale 2008 Pt. 7, Kotylar 2009b Pt. 7, Ochenrider 2010 Pt. 14, Thai 2010 Pt. 9, Kotlyar 2011 Pt. 4, AERS 6071719 | 19/M | IFX (3 infusions of 5 mg/kg /60 days), AZA (---/***^5^) |  | HSM | D (8) |
| **28** | Fowler 2010 Pt. 1, Kotlyar 2010 Pt. 13, Kotlyar 2011 Pt. 35, AERS 7415869, 7436216 | 19/M | AZA (2-2.5 mg/kg daily/2190 days) | Fatigue, Abdominal fullness | SM, L | D (4) |
| **29** | Ochenrider 2010 (index), AERS 7541028, 7553786, 7568547 | 18/M | 6MP (68.44 g/1825 days), MSL, PDE | Fatigue, dyspnea | F, SM; pallor | D (7) |
| **30** | Mackey 2007/2009 Pt. 6, Rosh 2007 Pt. 3, Shale 2008 Pt. 6, Kotylar 2009b Pt. 6, Ochenrider 2010 Pt. 12, Thai 2010 Pt. 10, Kotlyar 2011 Pt. 6, AERS 3915488* | 18/M | IFX (***^7^/210 days), AZA (---/1825 days), 6MP***^8^, PDE, BDE, alendronate sodium*, omeprazole* | Hepatitis | HSM, T; hepatitis | D (12-21*) |
| **31** | Mittal 2006, Mackey 2007 (body), Zeidan Pt. 3, Moran Pt. 4, Kotylar 2009a Pt. 2, Kotylar 2009b Pt. 18, Ochenrider 2010 Pt. 7, Kotlyar 2010 Pt. 4, Thai 2010 Pt. 3, Kotlyar 2011 Pt. 21, AERS 6803858 | 18/M | AZA (---/2190 days) | Swinging fever | F, HSM, P | D |
| **32** | AERS 6751796 | 18/M | AZA (1450 mg/39 days), 6MP (3900mg /39 days) |  |  | D (7) |
| **33** | Thayu 2005, Mackey 2007/2009 Pt. 4, Rosh 2007 Pt. 2, Zeidan 2007 Pt. 3, Shale 2008 Pt. 4, Kotlyar 2009a Pt. 6, Kotylar 2009b Pt. 4, Ochenrider 2010 Pt. 6, Kotlyar 2010 Pt. 1, Thai 2010 Pt. 7, Kotlyar 2011 Pt. 1, AERS 5653942 | 17/F | IFX, (20 infusions of 5 mg/kg each/***^5^), 6MP (93.53 g/1643 days), MSL (---/1825 days), PDE, HYC, metronidazole | Fever, malaise, abdominal pain | F, L, HSM, LFT | D (3) |
| **34** | Mackey 2007/2009 Pt. 2, Rosh 2007 Pt. 7, Shale 2008 Pt. 2, Kotylar 2009b Pt. 2, Ochenrider 2010 Pt. 9, Thai 2010 Pt. 13, Kotlyar 2011 Pt. 2, AERS 5907827* | 15/M | IFX (2450 mg in 13 infusions/487 days), AZA (---/730 days), 6MP*, MSL, PDE, ranitidine*, human growth hormone* |  | F, HSM, T, anemia | D (0.17) |
| **35** | AERS 7554658 | 13/M | 6MP, MSL, PDE |  |  | U |
| **36** | Mackey 2007/2009 Pt. 3, Rosh 2007 Pt. 1, Shale 2008 Pt. 3, Kotylar 2009b Pt. 3, Ochenrider 2010 Pt. 10, Thai 2010 Pt. 12, Kotlyar 2011 Pt. 3, AERS 5940088* | 12/M | IFX (6300 mg in 21 infusions/1764 days), AZA (---/1460 days), 6MP*, MSL, ciprofloxacin* | Rash | HSM, T | D (13) |
| **37** | Lemann 1998, Mackey 2007 (body), Zeidan 2007 Pt. 4, , Kotylar 2009b Pt. 20, Moran 2009 Pt. 1, Ochenrider 2010 Pt. 4., Thai 2010 Pt. 4, Kotlyar 2011 Pt. 23 | NR/NR | AZA (---/1460 days), PDE, CYC |  |  | D |
| INSUFFICIENT INFORMATION REPORTED TO CONFIRM | | | | | | |
| **38** | Mackey 2009 (in text), Kotlyar 2011 Pt. 30 | 18?/M | 6MP (3-5 years) |  |  | D |
| **39** | Mackey 2009 (in text), Kotlyar 2011 Pt. 31 | 33?/M | 6MP (3-5 years) |  |  | D |
| **40** | Grimpen 2009 | NR/NR | AZA, MSL, CS |  |  | U |
| **41** | Grimpen 2009 | NR/NR | AZA, MSL, CS |  |  | U |
| **42** | Falchook 2006/Falchook 2009, Pozadzides 2009 (text), Kotylar 2009b Pt. 23, Kotlyar 2010 Pt. 7, Kotlyar 2011 Pt. 26 | NR/NR | 6MP |  | SM | U |
| **43** | Mackey 2007 (in text), Ochenrider 2010 Pt. 13, Thai 2010 Pt. 5, Moran Pt. 5 | 30s/M | 6MP |  |  | D |
| **44** | AERS 6737750 | NR/M | IFX, AZA |  |  | U |
| **45** | AERS 6950655 | NR/F | IFX (9 infusions/---), PDL, paracetamol, pantoprazole |  |  | D |
| **46** | AERS 6924841 | ??/M | IFX (2 infusions) |  |  | U |

The table includes 37 patients identified as unique. The last 9 cases listed in the table may or may not represent unique patients. Taken together, the table contains at least 37 unique patients and possibly as many as 46 patients with Crohn’s disease who developed HSTCL.

**Abbreviations**:

**AERS,** Adverse Event Reporting System.

*Medications*: **6MP**, 6-mercaptopurine; **ADL**, adalimumab; **AZA**, azathioprine; **BDE**, budesonide; **BLS**, balsalazide; **CS**, corticosteroids unspecified; **CYC**, cyclosporine; **HYC**, hydrocortisone; **IFX**, infliximab; **MSL**, mesalamine; **NTL**, natalizumab; **PDE**, prednisone; **PDL**, prednisolone; **SLZ**, salazopyrine; **UST**, ustekinumab.

*Clinical Signs*: **F**, fever; **HSM**, hepatosplenomegaly; **L**, leucopenia; **LDH**, elevated lactate dehydrogenase levels; **LFT**, abnormal liver function tests; **N**, neutropenia; **P**, pancytopenia; **SM**, splenomegaly; **T**, thrombocytopenia

*Survival***: U**, unclear; **D**, death reported; **S**, survival.

**AERS cases are identified here by their case number.**

**Footnotes:**

*Additional data from matched AERS report(s).

** Age reported as 30 in AERS 6240979

***^1^According to Kotlyar 2011 [[26](#_ENREF_26)]

***^2^Conflicting information on # of infusions reported in published sources, using number from original report (Zeidan [[25](#_ENREF_25)]). Cumulative dose in mg remains the same.

***^3^Conflicting number of infusions reported in published sources

***^4^ Calculated using dose given in Mackey 2007/2009 [[18](#_ENREF_18), [19](#_ENREF_19)] as quantity used in a single infusion/injection

***^5^ Conflicting duration of treatment reported in published sources

***^6^Mackey 2007/2009 [[18](#_ENREF_18), [19](#_ENREF_19)] and Thai 2010 [[27](#_ENREF_27)] provided a range of 550-600 mg as the dose; cumulative dose calculated using 575 mg as the single infusion dose. Used the number of infusions reported in Thai 2010 [[27](#_ENREF_27)] instead of Mackey 2007/2009 [[18](#_ENREF_18), [19](#_ENREF_19)].

***^7^Conflicting number of infusions reported in published sources

***^8^ Medication not reported in Mackey 2007/2009 [[18](#_ENREF_18), [19](#_ENREF_19)]
